# Current Insights into Porcine Bocavirus (PBoV) and Its Impact on the Economy and Public Health

**DOI:** 10.3390/vetsci11120677

**Published:** 2024-12-22

**Authors:** Jelena Prpić, Tomislav Keros, Margarita Božiković, Magda Kamber, Lorena Jemeršić

**Affiliations:** 1 Croatian Veterinary Institute, Savska Cesta 143, 10000 Zagreb, Croatia; 2 Sanatio d.o.o., Nehajska 10, 10000 Zagreb, Croatia

**Keywords:** porcine bocavirus (PBoV), genome structure, classification, detection, public health concerns, economic impact

## Abstract

Effective control of animal infectious diseases is crucial for maintaining robust livestock production systems worldwide. Porcine meat constitutes approximately 35–40% of global meat production with the largest producers being China and the European Union (EU). Emerging viral pathogens in swine, like porcine bocavirus (PBoV), have not garnered significant attention, leaving their pathogenic characteristics largely unexplored. This review aims to bridge this knowledge gap by conducting a comprehensive analysis of the existing literature on PBoV. We explore the virus’s genome structure, discovery, classification, detection methods, pathogenesis, and its potential public health implications. Additionally, we discuss the distribution and economic impact of PBoV, which includes potential losses due to decreased productivity, increased veterinary costs, and trade restrictions. By highlighting the current state of knowledge, this review seeks to enhance the understanding of PBoV, thereby aiding in its prevention and control, and mitigating its economic impact on the swine industry.

## Simple Summary

Porcine Bocavirus (PBoV) is an emerging viral pathogen in swine that has not received significant attention despite its potential impact. This review aims to fill the knowledge gap by analyzing the existing literature on PBoV, including its genome structure, discovery, classification, detection methods, and pathogenesis. This review also explores the public health implications and economic impact of PBoV, such as decreased productivity, increased veterinary costs, and trade restrictions. By enhancing understanding of PBoV, this review seeks to aid in its prevention and control, thereby mitigating its economic impact on the swine industry.

## 1. Introduction

Pig meat is a major source of protein in the human diet, with a stable share of 35–40% of global meat production [1]. In Europe, the pig meat sector accounts for about 150 million reared pigs, representing nearly half of the total EU meat production [2]. The EU is the world’s second biggest producer of pork after China and the biggest exporter of pork and pork products, especially after the reduction in pork production in Asia, caused by African Swine Fever (ASF) [3]. However, even in countries of insufficient pig production, such as Croatia, high import can also be a high risk for disease introduction with a potentially severe economic impact. In Croatia, the members of the Croatian Association of Pork Producers are the largest producers of piglets, fattening pigs, and pig genetic material with a yearly production of about 700,000 fattening pigs and 30,000 sows [4], and according to the number of pigs and pig products, the Croatian contribution within the EU is below 1% [5]. However, 70 percent of Croatia’s pork meat market is covered by imports from other EU countries mostly from Germany, Spain, Hungary, the Netherlands, and Denmark [6] and is the 32nd largest importer of pig meat in the world.

Infectious agents, such as bacteria and viruses, are the main threats to production in intensive pig farms. Historically, many viral diseases, such as Porcine Reproductive and Respiratory Syndrome (PRRS), Porcine Circovirus-associated Diseases (PCVADs), and African Swine Fever and Foot and Mouth Disease (FMD), had a negative impact on the swine industry causing great economic losses [3,7,8,9,10,11]. Moreover, the zoonotic potential of viruses such as the causative agents of highly pathogenic avian influenza virus (HPAI), Severe Acute Respiratory Syndrome (SARS), and Middle East Respiratory Syndrome (MERS) have been shown to be of high risk for human health and should be identified in a timely manner. Recently, a number of small novel porcine DNA viruses have emerged globally, for example, torque teno sus virus, porcine bocavirus (PBoV), and parvoviruses. However, there is still a lack of knowledge regarding their biology, interspecies transmission, and potential pathogenicity. A newly described genetically unique class of viruses known as bocaparvovirus has been found in both humans and animals. One of the representatives of bocaviruses (BoVs) is an emerging pathogen that was recognized to cause a great negative impact on swine industry, namely PBoV. PBoV has currently been reported worldwide, mostly in weaning piglets. Since PBoV is common in both clinically and healthfully infected pigs, it is typically linked to co-infection with other viruses [12,13]. Therefore, it is important to understand PBoV and its pathogenic nature. Additionally, PBoV presents a serious risk to public health. A case study from Iran suggested that since PBoV was found in a three-year-old child who had an acute respiratory tract infection, the virus may be evolving as a human pathogen [14].

A study regarding PBoV prevalence in Croatia [15] reported its circulation among the Croatian domestic pig population. A conducted survey showed that PBoV is circulating among Croatian intensive pig farms and small family farms, and that PBoV might have been introduced by imported pigs. In order to protect a thriving pig industry and to facilitate early detection of the PoBV infection and its prevention and control, we aimed to summarize the current knowledge about PBoV, including the discovery, classification, genome structure, main diagnostic methods, pathogenesis, public health importance, distribution, and economic impact of PBoV.

## 2. Discovery and Classification

**Figure 1:**
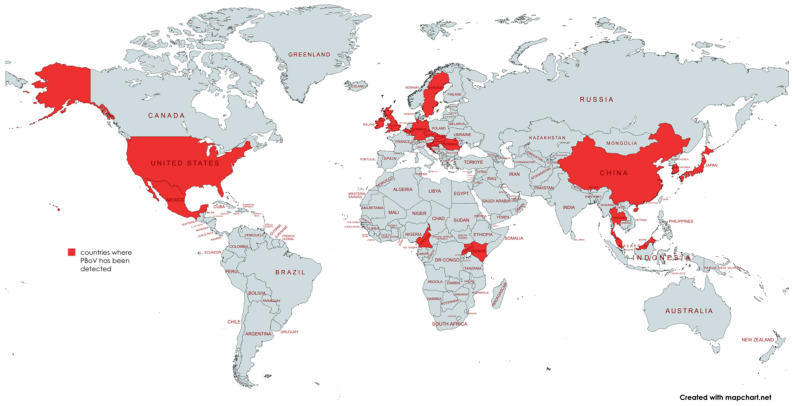
Geographical distribution of PBoV. Map is created with Mapchart [36].

Although BoVs were recognized in the early 1960s, PBoV was not isolated until 2009. Initially referred to as porcine boca-like virus (PBo-likeV), it was first detected in the lymph nodes of Swedish pigs suffering from postweaning multisystemic wasting syndrome (PMWS) [16,17]. In 2010, PBo-likeV was discovered in China and officially named PBoV or PBoV1 [18,19]. Since its discovery, PBoV has been reported globally (Figure 1; Table 1), including in Europe [15,20,21,22,23,24,25,26], Asia [27,28,29,30], North America [31,32], and Africa [33,34]. In recent studies, the detection of porcine bocavirus (PBoV) has been reported in various countries, highlighting its widespread presence and potential impact on swine health. Table 1 provides a comprehensive overview of the detection rates of PBoV in different countries, along with key details on the age of affected pigs and the sources of the samples collected. This information is crucial for understanding the epidemiology of PBoV and identifying the most vulnerable populations. Globally, the prevalence rate of PBoV was 46% in pigs without PMWS and 88% in pigs with PMWS [35]. In China, the prevalence rate of PBoV in stool samples of piglets was found to be 12.59%. Similarly, the prevalence rate of bocaviruses in clinical samples from one-month-old piglets was reported to be 5.77%. In the USA, studies have documented prevalence rates in sick pigs of approximately 43% and 59%. PBoV has been identified in various regions, including Africa. However, specific prevalence rates in Africa are not detailed in the sources. The virus is prevalent in both healthy and clinically infected pigs, often found in weaning piglets and associated with coinfections with other viruses [35]. The virus’s presence in these regions highlights its widespread nature and the potential for significant impacts on pig health and the swine industry across the continent. In Croatia, PBoV has been identified in pigs, contributing to the understanding of its epidemiology in Eastern Europe [15,35]. Studies have shown that the virus is present in both healthy and diseased pigs, indicating its pervasive nature and the need for ongoing surveillance and research to mitigate its impact on the swine industry [15,35].

BoVs belong to the genera *Bocaparvovirus*, family *Parvoviridae*, and subfamily *Parvovirinae* [37,38]. In contrast to other parvoviruses, BoVs contain a third open reading frame in the middle of the genome encoding for a highly phosphorylated non-structural protein, NP1, whose function has not yet been determined.

Their hosts are, as known to date, different mammalian species including primates (Table 2). Furthermore, the classification of PBoV within the broader context of the Bocaparvovirus genus is essential for understanding its relationship with other viruses in the Parvovirinae subfamily. Table 2 summarizes the viral species belonging to the genus Bocaparvovirus, as suggested by the International Committee on Taxonomy of Viruses (ICTV). This classification helps in elucidating the genetic and evolutionary relationships between PBoV and other related viruses, providing insights into their pathogenesis and potential cross-species transmission.

**Figure 2:**
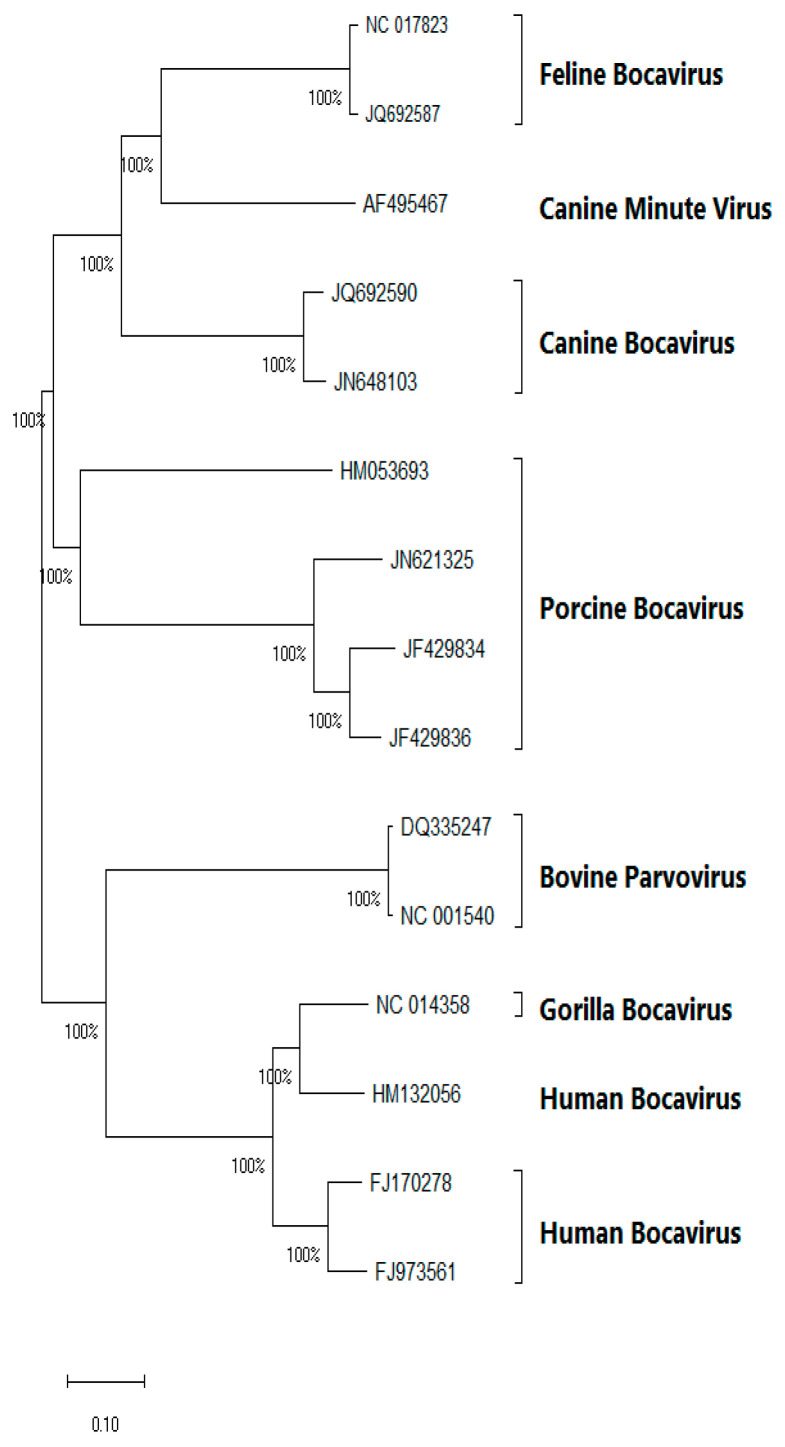
Phylogenetic tree of PBoV and related viruses based on the complete genome sequences. The tree was constructed using the Neighbor-Joining method with 1000 bootstrap replicates. Bootstrap values greater than 50% are shown at the branch nodes. The tree includes various BoV strains from different hosts, including humans, pigs, dogs, cats, cows, and gorillas. The scale bar represents the number of nucleotide substitutions per site. The phylogenetic tree reveals the relationships between PBoV and other bocaviruses. Notably, PBoV is closely related to other bocaviruses, including feline bocavirus and bovine bocavirus.

BoV strains that have been isolated from different hosts are closely related to PBoV. For instance, strains isolated from mink share 87% nucleotide similarity with PBoV (strain: PBoV-KU14; HQ223038, HQ291308, and KJ622366) and form a distinct clade within the PBoV clades [46,63]. Similarly, strains such as those isolated from bats [48,49,64], rodents [50,55,65], and Himalayan marmots [54] are also closely related to PBoV. These studies suggest that rodents, minks, bats, Himalayan marmots, and other species may have transmitted diseases to pigs. To date, however, there has been no reported experimental evidence of BoVs’ interspecies transmission. These investigations demonstrate the great genetic diversity and broad host adaptation of BoVs. Figure 2 illustrates the phylogenetic connections between porcine bocavirus (PBoV) and other bocaviruses.

## 3. Genome Structure

**Figure 3:**
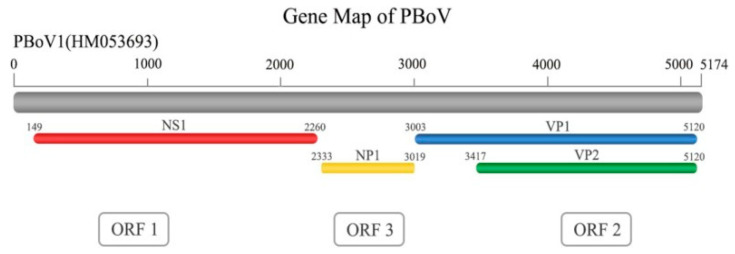
The genome structure of PBoV (adapted from [71]). The PBoV genome of approximately 5.3 bb contains three ORFs that encode for four proteins (NS1, NP1, VP1, and VP2).

PBoV is a nonenveloped single-stranded DNA virus which exhibits icosahedral symmetry [12,19] and has a diameter of 26–30 nm [66]. It is a linear, single-stranded DNA of 4–6 kb [12,19] and can have either positive or, more frequently, negative polarity [67] and contains three open reading frames (ORFs): ORF1, ORF2, and ORF3, respectively [53,68]. Specifically: ORF1 encodes a nonstructural protein 1 (NS1) which encodes a protein involved in ATPase and helicase activity and rolling circle replication [68]. Bovine parvovirus (BPV) and PBoV’s ORF-1 region are similar [60]. ORF2 encodes viral capsid proteins 1 and 2 (VP1/2), where VP1 includes the entire VP2 sequence and an additional N-terminal region [69]. ORF-2 encodes a protein linked to catalytic residues (the most conserved motif HDXXY), calcium-binding loop, and phospholipase A2 motifs (the most conserved motif YXGXG) necessary for parvovirus infectivity [12,19]. PBoV’s conserved motif, YXGXF, is distinct from other parvoviruses’ YXGXG motif [12,19]. The ORF-2 region of PBoV resembles canine minute virus (CMV) [61]. ORF3 encodes nuclear phosphoprotein 1 (NP1) of unclear function (Figure 3) [60].

The classification of PBoV into genotypes is based on nucleotide and amino acid alignments. Specifically, PBoV strains are grouped into different clades according to the sequences of the VP1 and VP2 genes [70]. The nucleotide sequence identity cutoff for distinguishing genotypes is generally between 78% and 81% [56]. Based on the VP1 and VP2 sequences, PBoV has been classified into different clades, including PBoV1, PBoV2, PBoV3, PBoV4, PBoV5, PBoV3C, PBoV-6V, and PBoV-7V. Additionally, PBoV has been proposed to be classified into three different groups: PBoV G1, PBoV G2, and PBoV G3 [71].

PBoV shows significant diversity, prevalence, and complexity among its strains. Phylogenetic and recombination analyses have revealed considerable variation in the genome sequences of different PBoV strains. For example, the NS1 gene of PBoV shares sequence identities with other bocaviruses, such as CMV, BPV, and human bocavirus (HBoV), ranging from 30% to 54.7% [71].

## 4. Main Diagnostic Methods

There are several PCR-based methods for rapid PBoV detection. For detection of the three groups of PBoV, conventional multiplex PCR can be successfully used. Multiplex PCR amplifies multiple DNA regions simultaneously using different sets of primers in a single PCR reaction. Single PCR assays and the multiplex PCR assay have equal sensitivity of 1.0 × 10^3^ genomic copies/lL for PBoV group 1, 4.5 × 10^3^ copies/lL for PBoV group 2, and 3.8 × 10^3^ copies/lL for PBoV group 3 [72]. It is a cost-effective, time-saving method which allows for the detection of multiple targets in one reaction. The main disadvantage is the requirement for the careful optimization of primer concentrations and annealing temperatures to avoid non-specific amplification. Loop-mediated isothermal amplification, or LAMP, was created for the quick, sensitive, and specific detection of PBo-likeV. LAMP amplifies DNA at a constant temperature using a set of four to six primers and a strand-displacing DNA polymerase. It has a stated detection limit of about ten copies per reaction and is about 100-times more sensitive than conventional PCR. Crucially, LAMP does not cross-react with major circulating swine viruses, including classical swine fever virus (CSFV), porcine parvovirus (PPV), porcine circovirus type 2 (PCV-2), and pig reproductive and respiratory syndrome virus (PRRSV) [73]. Primer design can be complex, and the method is less suitable for quantification compared to PCR. Targeting the VP1 gene, TaqMan-based real-time PCR (qPCR) has been utilized to detect and quantify ungulate bocavirus 2. TaqMan-based real-time PCR uses a fluorogenic probe to detect and quantify DNA during PCR amplification. This approach is quite specific and sensitive [68]. The specificity of the TaqMan PCR assay was evaluated using eight different reactions, which included Pbo-likeV, PRRSV, PCV2, pseudorabies virus (PRV), CSFV, Japanese encephalitis virus (JEV), and porcine torque tenovirus (PTTV). By comparing the signal intensity at various levels, Pbo-likeV was easily distinguished from other pig viruses. The sensitivity of the real-time PCR assay was evaluated by testing ten-fold serial dilutions of the DNA standards (2.00 × 10^1^ to 2.00 × 10^7^ copies) [40]. This method is quite expensive due to the cost of probes and requires specialized equipment. An EvaGreen-based multiplex real-time PCR approach was developed in order to identify and group the three BoVs groups (groups 1, 2, and 3) simultaneously in a single step [74]. This method utilizes EvaGreen dye and melting curve analysis to detect multiple targets in a single reaction. It is cost-effective, has high sensitivity, and allows for the detection of multiple pathogens. The main disadvantage is that this method requires careful optimization to avoid non-specific amplification and dye-related artifacts. Duplex nano PCR has been used to amplify and quantify PBoV and PRV simultaneously [75]. Duplex nano PCR combines nanopore sequencing with PCR to achieve high accuracy in detecting and quantifying DNA. The main advantages are its high accuracy, ability to sequence long reads, and suitability for complex samples. As a main disadvantage, the requirement for specialized equipment can be highlighted. SYBR Green-based duplex real-time PCR has been used for the detection of PBoV genotypes (3, 4, and 5) and porcine epidemic diarrhea virus (PEDV), with detection limits of 10 copies/lL for both viruses [76]. This method uses SYBR Green dye to detect and quantify DNA in real-time PCR, allowing for the simultaneous detection of two targets. It is cost-effective, simple to use, and suitable for high-throughput screening. This method is less specific than probe-based methods and can produce non-specific signals. Researchers in Sweden [16] implemented random amplification and high-throughput sequencing (HTS) to identify the PBo-likeV. These techniques are increasingly popular for discovering new viruses and addressing metagenomic challenges [40,66,77,78]. HTS allows for the sequencing of multiple samples simultaneously, generating large amounts of data quickly, but it is expensive and requires extensive data analysis and specialized equipment. Single-Primer Amplification (SISPA) for PBoV detection employs a single primer for sequence-independent PCR amplification of nucleic acids with unknown sequences. Researchers have successfully applied this method to amplify PBoV [19]. SISPA amplifies DNA using a single primer, allowing for the detection of unknown regions adjacent to known sequences. It is useful for genome walking and detecting unknown sequences but can produce non-specific amplification and requires careful optimization. Nested polymerase chain reaction has been used for the identification of PBoV from throat swab, fecal, and serum samples [69,78]. Nested polymerase chain reaction uses two sets of primers in two successive PCR reactions to increase specificity and sensitivity. It features high specificity, reduces non-specific amplification, and is suitable for detecting low-abundance targets, but it is more time-consuming and requires additional steps compared to conventional PCR. In the primer walking approach, researchers use specific primers in conjunction with a degenerate primer to target known BoV sequences [58]. The primer walking approach is a sequencing method that uses a series of overlapping primers to sequence long DNA fragments step-by-step. It is suitable for sequencing long DNA fragments and filling gaps in genome sequences, but it is time-consuming, labor-intensive, and requires careful primer design. The availability of additional sequences is essential for designing specific primers. Nucleic acid sequence-based amplification (NASBA), an isothermal amplification technique, assesses the polarity of the human BoV genome and analyzes uncharacterized gene transcription [67].

Indirect immunofluorescence tests have been developed to detect unknown isolated PBoV in primary porcinev kidney cell lines. Staining is observed in the cytoplasm and nuclei of infected cell cultures [21]. Additionally, monoclonal antibodies against PBoV 3 and 4 are produced for antigen-detecting ELISA [79]. An indirect enzyme-linked immunosorbent assay (ELISA) based on recombinant nucleoprotein 1 (NP1) investigates PBoV seroprevalence in China. Importantly, there is no cross-reactivity with antiserum against PRRSV, PCV-2, PPV, PEDV, or transmissible gastroenteritis virus (TGEV) [80].

## 5. Pathogenesis and Public Health Importance of PBoV

PBoV is quite common in the swine population and exhibits a high level of genetic diversity. However, its exact pathogenesis remains undetermined. Clinical evidence suggests that PBoV’s pathogenesis could be linked to direct disease manifestations [12]. Notably, PBoV has been detected in various tissues, indicating a wide tissue tropism [60]. Initial detection occurred in the lymph nodes of weaning pigs with post-weaning multisystemic wasting syndrome (PMWS) [16]. Subsequently, PBoV was found in respiratory tract samples and fecal samples from piglets [19]. In cases of gastrointestinal clinical signs, histopathological alterations (microscopic lesions and villous atrophy) were primarily found in the jejunum, ileum, and duodenum [81]. Additionally, the pathogenic role of PBoV has been highlighted by its isolation from piglets suffering from encephalomyelitis [24]. A study also identified a new porcine parvovirus (PPV4), similar to PBoV, in lung lavage from a diseased pig co-infected with PCV-2. Inoculation of colostrum-deprived pigs with lung, lymph nodes, spleen, and heart tissue homogenates resulted in respiratory disease and euthanasia.

The pathophysiology of PBoV is still unknown, despite its great frequency in the swine population and considerable genetic variety [31]. While PBoV has been detected in various tissues, including lymph nodes, spleen, and tonsils, it remains unclear which specific cells in pigs contribute to its replication and dissemination throughout the body [30]. Notably, PBoV has also been found in saliva and serum [22,28,82]. In a study conducted with dogs in Thailand, researchers reported that most viruses from the *Parvoviridae* family replicate in mitotically active cells, such as intestinal crypt epithelial cells. Examination of tissue samples from the small intestine revealed nuclear signals for canine bocavirus 2 primarily in enterocytes located at villus tips and crypts. Transmission electron microscopy showed electron-dense icosahedral viral particles measuring approximately 20 nm in diameter, creating large intranuclear inclusion bodies within apical small intestinal enterocytes [83]. These findings shed light on canine bocavirus infections’ pathogenicity and may have implications for other BoVs isolated from different animals. Interestingly, rats with a parvovirus infection [26] generally remain healthy. But, occasionally, pregnant rats experimentally inoculated with parvovirus exhibit growth retardation and fetal loss. Despite these insights, cell lines for BoVs’ isolation have not yet been reported.

For PBoV propagation, various cell types have been used, for example human embryonic kidney epithelial cells (HEK293T), monkey kidney cells (MARC-145), porcine kidney cells (PK-15), porcine testicular cells, and porcine alveolar macrophages. However, despite these efforts, no successful propagation has been reported [59].

During a diarrheal disease outbreak in Belgium, researchers attempted to isolate recombinant enterovirus-torovirus and BoV from porcine testicular cells and primary porcine kidney epithelial cells. No virus was detected in passaged cells, despite the fact that after two days a cytopathic effect was seen in pig kidney epithelial cells [25].

Another study conducted in Northern Ireland reported that after four passes in cultured primary swine kidney cells, fecal suspensions and homogenates from the small intestines of 6-week-old piglets showed a cytopathic effect. Interestingly, PCR and RT-PCR did not find porcine enterovirus types 1, 2, or 3, porcine adenovirus, porcine reovirus, PCV1, PCV-2, or PPV. Successful BoV proliferation in a primary cell line was originally reported in this study [21]. However, due to the virus’s inability to be cultured in cell lines and its common coexistence with other circulating swine enteric viruses, its precise role in disease progression remains unclear. The association of BoV’s with other pathogenic enteric viruses has perpetually confounded its nature. Additionally, the dual involvement of BoVs in respiratory tract infections and gastrointestinal tract infections raises questions about whether they primarily act as respiratory or enteric pathogens, especially in humans, pigs, and other animals. Unfortunately, the lack of an experimental animal model has hindered investigations into the pathogenic nature of PBoV.

The fecal–oral route may serve as the primary transmission pathway for PBoV, similar to other enteric pathogens. Diarrheal feces, vomitus, and other contaminated fomites (such as feeds and transportation vehicles) are important sources of transmission. As a nonenveloped virus, PBoV is resistant to severe environmental conditions and helps transmit other parvoviruses widely [27]. In 2007, human BoVs were detected in 81% of raw sewage samples from several US states [84], highlighting the robustness of BoVs. PBoV can spread across the swine population and induce subclinical infections in pigs once it is introduced into swine herds. In order to determine whether aerosolized PBoV is infectious, further investigation is needed.

Human BoV human parvovirus 4 and parvovirus B19, both belonging to the *Parvoviridae* family, are associated with human diseases [85,86]. Human BoVs have a global presence [87]. The first documented case of human BoV occurred in a child’s respiratory tract in Sweden in 2005, four years before BoV was identified in swine [52]. BoVs have been found in both the respiratory tract [53,84,88] and gastrointestinal tract [89], as well as in cancer patients [90]. These viruses have also been detected in healthy individuals. However, their role in human health remains unknown, since they may act as true pathogens, opportunistic pathogens, and commensal flora. The description of PBoV in a three-year-old kid suffering from an acute respiratory tract infection in an Iranian case study from 2018 [14] raises the possibility that PBoV is evolving as a human pathogen. The risk of interspecies transmission of PBoV to human subjects can arise when xenotransplantation is carried out [86]. Therefore, given the growing concern in regard to the pathogenic role of PBoV, it is recommended to screen and analyze the transmission risk of ssDNA viruses to prevent post-transplantation complications.

The possibility of interspecies transmission from rats and pigs to humans requires more research [49]. Significant public health concerns are raised by the vast host range, wide tissue tropism, and growing pathogenic potential of BoVs.

## 6. Economic Impact and Prevention

PBoV has significant economic implications for the swine industry and food industry. It can lead to reduced growth rates and increased mortality in piglets, which directly impacts productivity. Infected pigs often exhibit symptoms such as diarrhea and respiratory issues, leading to lower weight gain, higher feed conversion ratios, and increased mortality rates, all of which negatively impact farm profitability [35]. For instance, studies have shown that co-infection with PBoV and other pathogens, such as PRRSV, can exacerbate these effects, leading to even greater economic losses [91]. A study on the impact of PBoV and PRRSV co-infection found that pigs with the worst production performance had significantly higher levels of co-infection, resulting in increased mortality and lower weight gain [92]. The study highlighted that infected pigs showed a reduction in daily live weight gain (DLWG) and deteriorating feed conversion ratios (FCRs), which are critical indicators of production efficiency [92]. Managing PBoV involves substantial veterinary expenses. Farmers need to invest in diagnostic tests, treatments, and preventive measures to control the spread of the virus. This includes costs for medications, vaccines, and biosecurity measures [70]. Outbreaks of PBoV can lead to trade restrictions and export bans, especially if the virus is detected in countries that are major exporters of pork. These restrictions can result in substantial financial losses for producers and affect the overall market dynamics, as well as prices of meat [35]. Overall, the economic impact of PBoV is multifaceted, affecting productivity, increasing costs, and disrupting trade. Addressing these challenges requires coordinated efforts in disease management and prevention.

Preventing PBoV outbreaks involves several key strategies. Implementing strict biosecurity protocols is crucial. This includes controlling access to farms, disinfecting equipment and vehicles, and ensuring that workers follow hygiene practices to prevent the introduction and spread of the virus [71]. While there is no specific vaccine for PBoV yet, keeping pigs vaccinated against other common diseases can help maintain overall herd health, reducing the risk of secondary infections that can exacerbate PBoV symptoms [35]. Conducting regular health checks and diagnostic tests can help detect PBoV infection in an early stage of introduction to the farm. Early detection allows for timely intervention, reducing the spread of the virus within the herd [90]. Isolating new or returning animals for a period before introducing them to the main herd can prevent the introduction of PBoV from external sources [71]. Ensuring proper nutrition, reducing stress, and maintaining clean living conditions can strengthen the pigs’ immune systems, making them less susceptible to infections [35]. Keeping farm staff informed about PBoV and its prevention is essential. Regular training sessions on biosecurity and disease management can help maintain high standards of farm hygiene and health [91]. By combining these strategies, farmers can significantly reduce the risk of PBoV outbreaks and protect their herds from this economically impactful virus.

International and national legal provisions play a crucial role in regulating the export of pigs to prevent the spread of diseases, including PBoV. The movement of porcine animals within the EU is regulated by Regulation (EU) 2016/429 of the European Parliament and of the Council (Animal Health Law, AHL) on transmissible animal diseases. This regulation aims to prevent the spread of animal diseases by setting strict biosecurity measures and health requirements for the transport of porcine animals [93]. Additionally, Commission Delegated Regulation (EU) 2020/688 lays down specific health guarantees for the movement of porcine animals to ensure they do not pose a significant risk of spreading diseases [93]. The UK has implemented restrictions on the import of fresh porcine meat and meat products from areas within the EU affected by African Swine Fever (ASF). These restrictions are outlined in various health certificates and regulations, which require that porcine meat products from ASF-affected areas undergo specific treatments to mitigate the risk of disease transmission [94]. International trade rules, such as those enforced by the World Trade Organization (WTO), allow for import bans to be applied to specific regions or establishments affected by disease outbreaks. This principle, known as “regionalization,” ensures that trade can continue from disease-free regions within exporting countries, thereby preventing the spread of diseases like PBoV [95]. These regulations highlight the importance of stringent health measures and biosecurity protocols in the export of pigs to prevent the spread of diseases, including PBoV. By adhering to these legal provisions, countries can safeguard their livestock industries and protect public health.

## 7. Conclusions

BoV, an emerging pathogen with a broad host range and wide tissue tropism, has challenged researchers over the past decade. Despite numerous attempts to isolate this virus in cell culture, success has remained elusive. Consequently, an organ co-culture system may prove beneficial for BoV propagation. Such a system could shed light on whether the virus predominantly targets the respiratory or gastrointestinal tract or other tissue systems.

Numerous studies from different countries have connected PBoV to swine coinfecting diseases such as rotavirus A, PEDV, PRRSV, and CSFV. But even ten years after its description, we are still unsure if PBoV is a real pathogen or just an opportunistic one. To address this, single-virus isolation could be employed in animal infection models. Direct proof of the pathophysiology of PBoV is also essential.

Complex evolutionary pathways, interspecies transmission, and recombination sites have been uncovered by phylogenetic analysis of PBoV isolated from mice and minks. To clarify interspecies transmission across several host species, more investigation is required. Given its potential impact, BoV warrants attention as a significant public health problem. An Iranian clinical case [14] serves as a reminder of the importance of understanding emerging pathogens like PBoV. Our contributions to PBoV research will aid in prevention, control, and mitigating its economic consequences.

The economic ramifications of PBoV distribution are complex. Although the full pathogenic potential of PBoV remains unclear, its interaction with other viral infections can intensify disease severity in pigs, leading to higher rates of illness and death. This situation can cause substantial financial losses for the swine industry due to reduced productivity, increased veterinary expenses, and the necessity for stricter biosecurity protocols. Additionally, the detection of PBoV in pig populations can impact international trade, as countries might enforce restrictions on the import and export of pigs and pork products from affected areas. Such measures can limit market access and result in financial setbacks for pig farmers and associated industries.

In summary, the extensive spread of PBoV and its possible role in disease outbreaks highlight the critical need for ongoing research, vigilant monitoring, and the development of effective control strategies to safeguard the global swine industry and minimize economic losses.

## Figures and Tables

**Table 1:** Detection of PBoV in various countries: key details on age of affected pigs and sample sources.

Country	Year of Identification	Sample Source	Age Categories	References
Sweden	2009	Lymph nodes	weaners	[16,17]
China	2010	Fecal samples	weaners	[18]
USA	2010	Lung, lymph nodes, spleen	unknown	[31]
Ireland	2011	Spleen	piglets, weaners, sows	[21]
Romania	2011	Lymph nodes, lung, kidney, liver, spleen, tonsil	piglets, yearlings, adults wild boar	[20]
Hungary	2012	Fecal samples, blood serum samples, organ tissues, fetuses, semen	weaners	[22]
Croatia	2013	Fecal samples	fatteners	[15]
Cameroon	2013	Fecal samples	piglets	[33]
UK	2014	Lung, liver, kidney, spleen, lymph nodes, serum samples	unknown	[26]
Thailand	2014	Tonsil	rearing pigs	[27]
Korea	2014	Serum and fecal samples, saliva	weaners	[28]
Czech Republic	2014	Lymph nodes, liver, spleen	piglets, weaners, fatteners	[23]
Slovakia	2014	Lymph nodes, liver, spleen	piglets, weaners, fatteners	[23]
Mexico	2015	Serum samples	unknown	[32]
Germany	2016	Lung, lymph nodes	weaners	[24]
Japan	2017	Tonsil	unknown	[29]
Uganda	2017	Fecal samples	unknown	[34]
Kenya	2017	Fecal samples	unknown	[34]
Slovenia	2017	Fecal samples	unknown	[15]
Belgium	2018	Fecal samples	fatteners	[25]
Malaysia	2018	Mesenteric lymph node, submandibular lymph node, inguinal lymph node, spleen, tonsil, lung, kidney, liver	piglets, weaners	[30]

**Table 2:** Summary of viral species belonging to the genus *Bocaparvovirus*, subfamily *Parvovirinae*, and family *Parvoviridae*, as suggested by the ICTV [26].

Host	Species	Virus Name	Abbreviation	References
family *Canidae*	*Carnivore bocaparvovirus* 1	Canine minute virus; minute virus of canines	CnMV; MVC	[39,40]
	*Carnivore bocaparvovirus* 2	Canine bocavirus 2	CBoV2	[41]
	*Carnivore bocaparvovirus* 7	Canine bocavirus 3	CBoV3	[42]
family *Felidae*	*Carnivore bocaparvovirus* 3	Feline bocavirus 1	FBoV1	[43]
	*Carnivore bocaparvovirus* 4	Feline bocaparvovirus 2	FboV2	[44]
	*Carnivore bocaparvovirus* 5	Feline bocaparvovirus 3	FBoV3	[45]
family *Mustelidae*	*Carnivore bocaparvovirus* 6	Mink bocavirus 1	MiBoV1	[46]
family *Vespertilionidae*	*Chiopteran bocaparvovirus* 1	Myotis myotis (bat) bocavirus 1	BtBoV1	[47]
	*Chiopteran bocaparvovirus* 2	Bat bocavirus WM40	BtBoVwM40	[48]
	*Chiopteran bocaparvovirus* 3	Bat bocavirus XM30	BtBoVxm30	[49]
	*Chiopteran bocaparvovirus* 4	Miniopterus schreibersii bat bocavirus	BtBoV2	[40]
	*Chiopteran bocaparvovirus* 5	Rousettus leschenaultia bocaparvovirus 1	RIBoV	[50]
family *Leporidae*	*Lagomorph bocaparvovirus* 1	Rabbit bocaparvovirus	RBoV	[51]
family *Otariidae*	*Pinniped bocaparvovirus* 1	California sea lion bocavirus 1	CsIBoV1	[40]
	*Pinniped bocaparvovirus* 2	California sea lion bocavirus 3	CsIBoV3	[40]
family *Hominidae*	*Primate bocaparvovirus* 1	Human bocavirus 1 and 3	HBoV1, 3	[52]
	*Primate bocaparvovirus* 2	Human bocavirus 2 and 4	HBoV2, 4	[53]
family *Cercopithecidae*	*Primate bocaparvovirus* 3	Macca mulatta bocaparvovirus	MmBoV	[54]
family *Muridae*	*Rodent bocaparvovirus* 1	Rat bocavirus	RBoV	[50]
	*Rodent bocaparvovirus* 2	Murine bocavirus	MuBoV	[55]
family *Bovidae*	*Ungulate bocaparvovirus* 1	Bovine parvovirus 1	BPV1	[56]
	*Ungulate bocaparvovirus* 6	Bovine bocaparvovirus 2	BBoV2	[57]
family *Suidae*	*Ungulate bocaparvovirus* 2	Porcine bocavirus 1	PBoV1	[16]
	*Ungulate bocaparvovirus* 3	Porcine bocavirus SX	PBoVsx	[58]
	*Ungulate bocaparvovirus* 4	Porcine bocavirus H18	PBoVh18	[59]
	*Ungulate bocaparvovirus* 5	Porcine bocavirus 3	PBoV3	[60]
family *Camelidae*	*Ungulate bocaparvovirus* 7	Dromedary camel bocaparvovirus 1	DBoV1	[61]
	*Ungulate bocaparvovirus* 8	Dromedary camel bocaparvovirus 2	DBoV2	[61]
	*Ungulate bocaparvovirus* 9	Vicugna pacos bocaparvovirus	VpBoV	[62]

## Data Availability

No new data were created or analyzed in this study. Data sharing is not applicable to this article.
